# Evaluation of convolutional neural networks for herbicide susceptibility-based weed detection in turf

**DOI:** 10.3389/fpls.2023.1096802

**Published:** 2023-02-01

**Authors:** Xiaojun Jin, Teng Liu, Patrick E. McCullough, Yong Chen, Jialin Yu

**Affiliations:** ^1^ College of Mechanical and Electronic Engineering, Nanjing Forestry University, Nanjing, Jiangsu, China; ^2^ Peking University Institute of Advanced Agricultural Sciences / Shandong Laboratory of Advanced Agricultural Sciences at Weifang, Weifang, Shandong, China; ^3^ Department of Crop and Soil Sciences, University of Georgia, Griffin, GA, United States

**Keywords:** deep learning, convolutional neural networks, weed detection, herbicide susceptibility, precision herbicide application

## Abstract

Deep learning methods for weed detection typically focus on distinguishing weed species, but a variety of weed species with comparable plant morphological characteristics may be found in turfgrass. Thus, it is difficult for deep learning models to detect and distinguish every weed species with high accuracy. Training convolutional neural networks for detecting weeds susceptible to herbicides can offer a new strategy for implementing site-specific weed detection in turf. DenseNet, EfficientNet-v2, and ResNet showed high F_1_ scores (≥0.986) and MCC values (≥0.984) to detect and distinguish the sub-images containing dollarweed, goosegrass, old world diamond-flower, purple nutsedge, or Virginia buttonweed growing in bermudagrass turf. However, they failed to reliably detect crabgrass and tropical signalgrass due to the similarity in plant morphology. When training the convolutional neural networks for detecting and distinguishing the sub-images containing weeds susceptible to ACCase-inhibitors, weeds susceptible to ALS-inhibitors, or weeds susceptible to synthetic auxin herbicides, all neural networks evaluated in this study achieved excellent F_1_ scores (≥0.995) and MCC values (≥0.994) in the validation and testing datasets. ResNet demonstrated the fastest inference rate and outperformed the other convolutional neural networks on detection efficiency, while the slow inference of EfficientNet-v2 may limit its potential applications. Grouping different weed species growing in turf according to their susceptibility to herbicides and detecting and distinguishing weeds by herbicide categories enables the implementation of herbicide susceptibility-based precision herbicide application. We conclude that the proposed method is an effective strategy for site-specific weed detection in turf, which can be employed in a smart sprayer to achieve precision herbicide spraying.

## Introduction

Turfgrass is widely grown in urban landscapes, including institutional and residential lawns, parks, or athletic fields (Potter and Braman, 1991). The total turfgrass area in the United States is 163,812 km^2^, which accounts for approximately 1.9% of the whole terrestrial land of the country (Milesi et al., 2005). Weed control is a challenging task for turfgrass management. Weeds compete with the turfgrass for sunlight, moisture, and soil nutrients, reducing turf aesthetics, surface quality, and functionality (Hamuda et al., 2016; Liu and Bruch, 2020). Weed management in turfgrass landscapes has relied heavily on broadcast herbicide application (McElroy and Martins, 2013), although weeds almost always present in non-uniform and patchy distributions (Dai et al., 2019; Yu et al., 2019a). Excessive application of synthetic herbicides could potentially pose a risk to human health and cause environmental pollution (Slaughter et al., 2008; Dai et al., 2019; Yu et al., 2019b; Mennan et al., 2020). Moreover, the application of synthetic herbicides represents a significant variable cost in turf weed management (Davis and Frisvold, 2017). These concerns have led to legal regulations regarding herbicide usage in several countries. For example, the European Union encourages spot-spraying to reduce the herbicide input (Busey, 2003; Marchand and Robin, 2019). Additionally, spot-spraying could effectively minimize the amount reaching off-target areas (Melland et al., 2016). In the United States, Environmental Protection Agency has proposed a series of measures, including prohibiting aerial applications for all atrazine labels to reduce their chance of runoff from the managed fields (Pimentel and Burgess, 2012; McCullough et al., 2015).

Site-specific weed management is a promising solution for sustainable weed control (Chen et al., 2022). Precision spraying a particular type or volume of herbicide onto susceptible weed species can significantly reduce herbicide input and weed control costs (Liakos et al., 2018). Site-specific weed management relies on the accurate identification and localization of weeds (Fennimore et al., 2016; Wang et al., 2019). Previous researchers explored various visual characteristics, such as color (Tang et al., 2016), morphological (Perez et al., 2000), hyper- or multi-spectral (Pantazi et al., 2016; Jiang et al., 2020), and textural features (Bakhshipour et al., 2017), for weed detection. However, crops and weeds may exhibit similar visual characteristics, thus detection and classification of weeds in crops are inherently challenging (Hasan et al., 2021). In turf, weed detection is challenging due to the presence of a variety of weed species growing with turfgrass.

In recent years, deep learning, a subfield of artificial intelligence, has demonstrated remarkable capability in speech recognition (Hinton et al., 2012; LeCun et al., 2015), natural language processing (Collobert and Weston, 2008; Collobert et al., 2011), and computer vision (Gu et al., 2018; Shi et al., 2020; Zhou et al., 2020). Deep learning technologies exhibit a tremendous ability to learn representations from raw data and extract complex features from images with a high accuracy level (Jordan and Mitchell, 2015; He et al., 2020; Yang et al., 2022a). Moreover, the improvements in graphics processing units (GPUs) have facilitated the use of deep convolutional neural networks (Bao et al., 2017; Bao et al., 2021; Ngo et al., 2021). Recent studies have investigated the feasibility of using deep learning in various agricultural domains, including plant disease detection (Martinelli et al., 2015; Saleem et al., 2019), crop yield prediction (Khaki and Wang, 2019; Van Klompenburg et al., 2020), plant phenotyping (Atefi et al., 2021; Zhang et al., 2022), and weed detection (Jin et al., 2021; Peng et al., 2022; Razfar et al., 2022). For example, Abbas et al. proposed a deep learning-based method for tomato disease detection. The trained neural network achieved a best 5-class classification accuracy of 99.51 (Abbas et al., 2021). Subeesh et al. compared four convolutional neural networks, including AlexNet, GoogLeNet, InceptionV3, and Xception for detecting various weeds growing in bell peppers (*Capsicum annum* L.) and found InceptionV3 achieved the highest accuracy (97.7%) (Subeesh et al., 2022). For image-based weed detection and discrimination, previous findings suggest that deep learning methods generally outperform other methods (Fennimore et al., 2016; Kamilaris and Prenafeta-Boldú, 2018).

Several studies have investigated the use of image classification or object detection neural networks for detecting and distinguishing various weed species in turfgrass (Yu et al., 2019a; Yu et al., 2019b; Yu et al., 2019c; Yu et al., 2020). Jin et al. demonstrated that VGGNet effectively detected and distinguished dallisgrass (*Paspalum dilataum* Poir.), purple nutsedge (*Cyperus rotundus* L.), and white clover (*Trifolium repens* L.) growing in bermudagrass [*Cynodon dactylon* (L.) Pers.] turf, while RegNet is well-performed in discriminating common dandelion (*Taraxacum officinale* Web.) (Jin et al., 2022). In another study, Yu et al. developed effective deep convolutional neural networks to detect weeds in turf. The authors reported that the image classification neural network VGGNet reliably classified broadleaf and grassy weeds growing in bermudagrass turf. In addition, the object detection neural network DetectNet achieved high overall accuracy at detecting cutleaf evening-primrose (*Oenothera laciniata* Hill) growing in bahiagrass (*Paspalum notatum* Flugge) (Yu et al., 2019b; Yu et al., 2019c).

Different weed species exhibit varying susceptibility to a particular herbicide category (McElroy and Martins, 2013; Yu et al., 2018). For example, acetolactate synthase (ALS)-inhibiting herbicides generally provide a narrow weed control spectrum (Yu and Boyd, 2018); ACCase-inhibiting herbicides are only effective for controlling grassy weeds (McElroy and Martins, 2013); nonselective herbicides, such as glyphosate and glufosinate, could nonselectively control all weeds (Johnson, 1977); and synthetic auxin herbicides, such as 2,4-D, dicamba, and MCPA, are only effective for controlling broadleaf weeds (McElroy and Martins, 2013). Therefore, precision spraying herbicides based on the susceptibility of different weed species to the herbicides can significantly reduce herbicide input and improve herbicide use efficiency. Although deep learning has been well-performed in weed detection and discrimination, previous studies have generally focused on distinguishing different weed species and did not establish a direct connection between weeds and herbicides. Moreover, a variety of weed species with comparable plant morphological characteristics may be found in turfgrass, thus it is difficult for the deep learning models to detect and distinguish every weed species with high accuracy. In the present research work, in addition to the detection and discrimination of individual weed species, different weed species growing in bermudagrass turf were grouped according to their susceptibility to herbicides, and weeds were detected and distinguished by herbicide categories. The proposed method would allow precision herbicide application based on susceptibility and thereby effectively reduce herbicide input while achieving the same level of weed control as the broadcast herbicide application. The objectives of this paper were to (1) investigate the feasibility of utilizing deep learning for herbicide susceptibility-based weed detection in bermudagrass turf, and 2) evaluate and compare the performance of different convolutional neural networks for distinguishing individual weed species.

## Materials and methods

### Overview

The image classification convolutional neural networks, including DenseNet (Huang et al., 2017), EfficientNet (Tan and Le, 2019), and ResNet(He et al., 2016), were selected for evaluating the feasibility of using the convolutional neural networks for detecting and distinguishing individual weed species growing in bermudagrass turf or detecting and distinguishing weeds susceptible to herbicides. DenseNet is a convolutional neural network that computes dense and multi-scale features from the convolutional layers. For each layer, it obtains additional inputs from all preceding layers and passes on its feature maps to all subsequent layers. EfficientNet uses a set of fixed scaling coefficients to uniformly scales all dimensions of depth, width, and resolution in a principled way. The EfficientNet achieves state-of-the-art accuracy with 10× better efficiency by utilizing this novel scaling method. ResNet introduced the concept of residual learning. It employs an identity-based skip connection in each residual unit. ResNet eases the flow of information across units and thus can gain accuracy from very deep networks. In this study, these three convolutional neural networks were trained and evaluated with the ultimate goal of site-specific herbicide application.

### Image acquisition

The training images of crabgrass (*D.igitaria ischaemum* L.), dollarweed (*Hydrocotyle* spp.), old world diamond-flower (*Hedyotis cormybosa* L.), and tropical signalgrass [*Urochloa distachya* (L.) T.Q. Nguyen] were acquired at several golf courses in Bradenton (27.49°N, 82.47°W), Riverview (27.86°N, 82.32°W), Sun City (27.71°N, 82.35°W), and Tampa (27.95°N, 82.45°W), Florida, while the testing images were acquired at several golf courses and institutional lawns in Lakeland, Florida (28.03°N, 81.94°W). The training images of goosegrass (*Eleusine indica* L.) and Virginia buttonweed (*Diodia virginiana* L.) growing in bermudagrass turf were acquired at the University of Georgia Griffin Campus in Griffin, Georgia, United States (33.26°N, 84.28°W), while the testing images were acquired at several golf courses in Peachtree City, Georgia, United States (33.39°N, 84.59°W). The training images of purple nutsedge were acquired at sod farms in Jiangning District, Nanjing, Jiangsu, China (31.95°N, 118.85°E), while the testing images were acquired at sod farms in Shuyang, Jiangsu, China (34.12°N, 118.79°E). The training and testing images of crabgrass, dollarweed, goosegrass, old world diamond-flower, tropical signalgrass, and Virginia buttonweed were captured multiple times from April to November 2018 using a digital camera (DSC-HX1, SONY^®^, Cyber-Shot Digital Still Camera, SONY Corporation, Minato, Tokyo, Japan). The training and testing images of purple nutsedge were captured in spring 2021 using a digital camera (Panasonic^®^ DMC-ZS110, Xiamen, Fujian, China). The original resolution of the training and testing images was 1,920 × 1,080 pixels. To enrich the diversity of the training dataset, images were captured under various illumination conditions, including partly cloudy, cloudy, or sunny days.

### Training and testing

**Figure 1:**
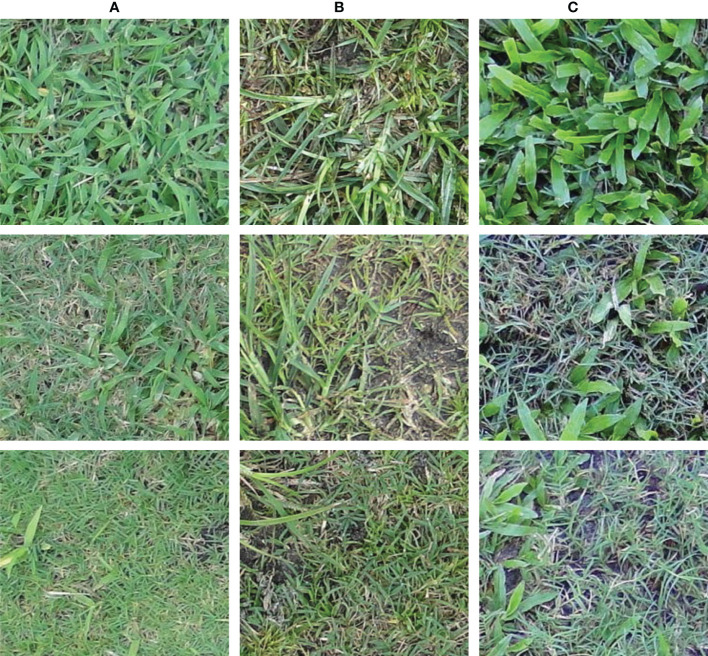
The training and testing sub-images of crabgrass **(A)**, goosegrass **(B)**, and tropical signalgrass **(C)** at different growth stages and densities.

**Figure 2:**
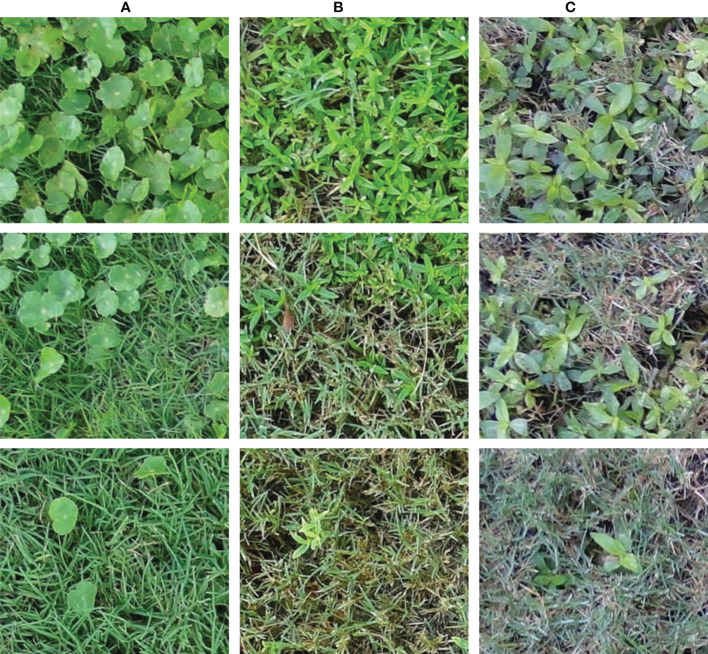
The training and testing sub-images of dollarweed **(A)**, old world diamond-flower **(B)**, and Virginia buttonweed **(C)** at different growth stages and densities.

**Figure 3:**
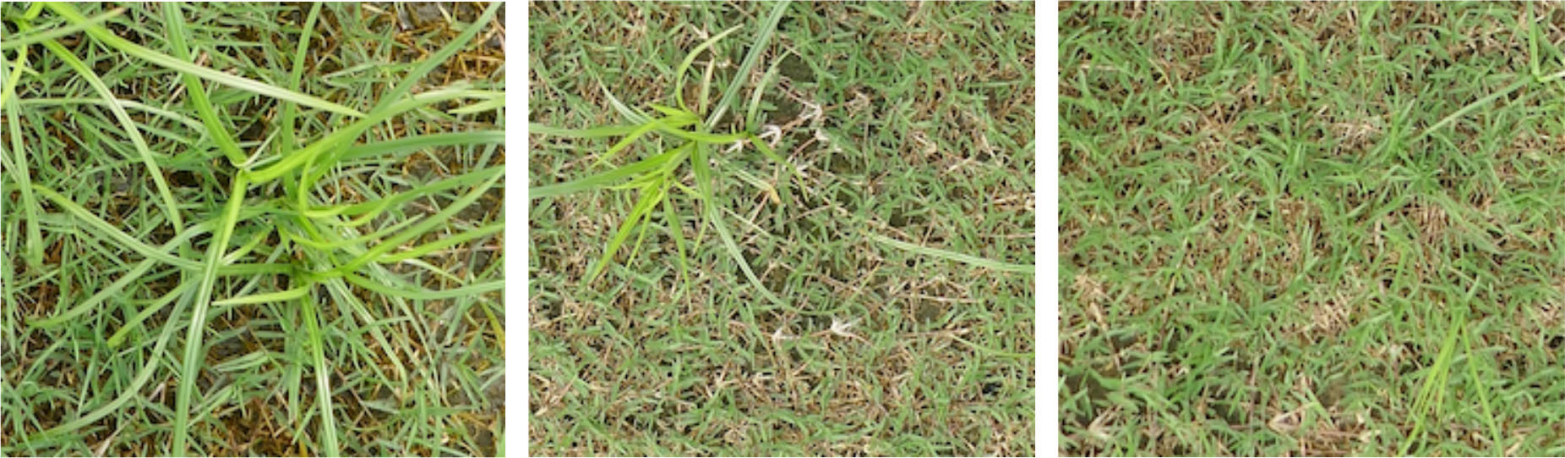
The training and testing sub-images of purple nutsedge at different growth stages and densities.

**Figure 4:**
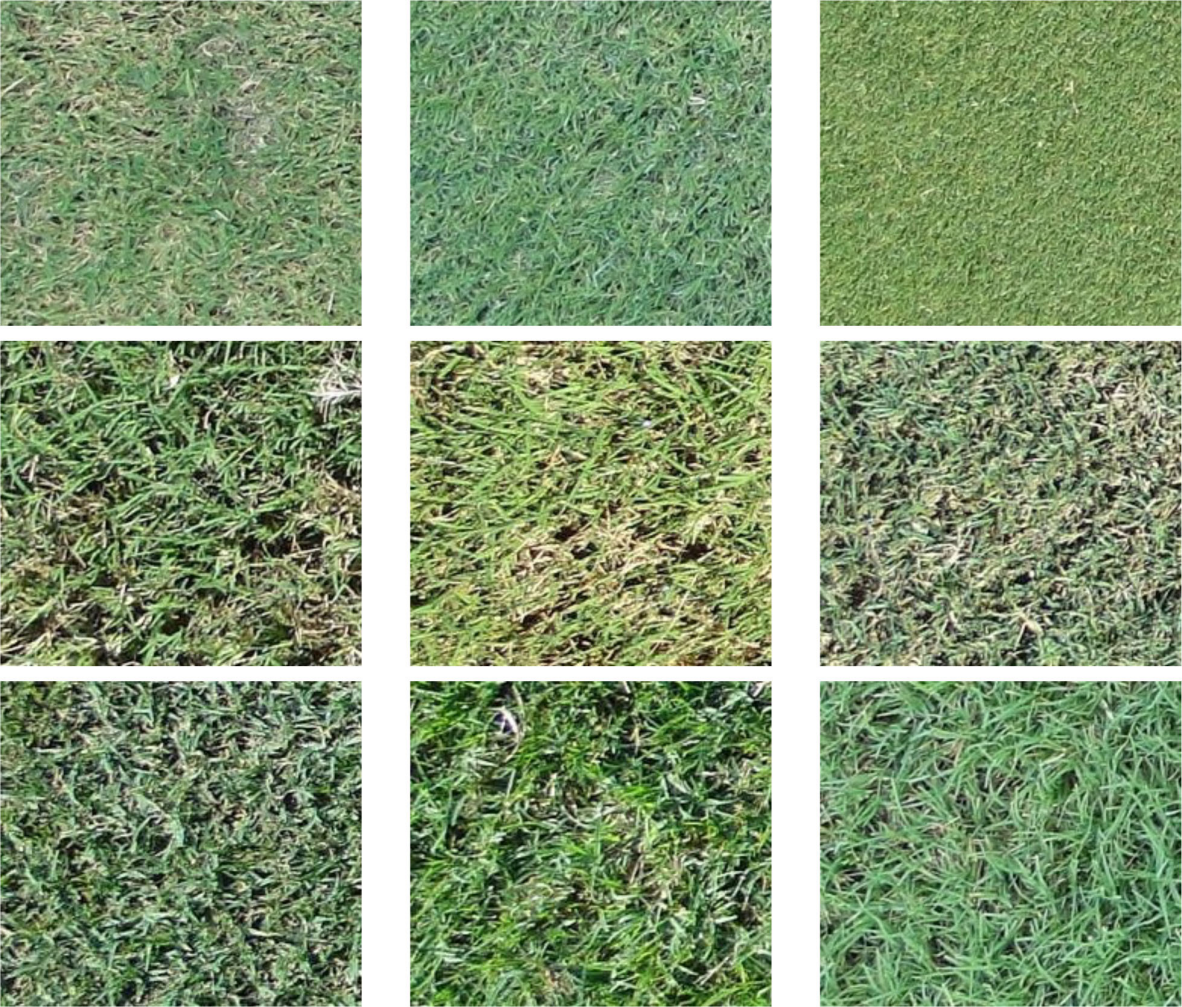
The training and testing sub-images of bermudagrass at different turfgrass management regimes, mowing heights, and surface conditions.

**Figure 5:**
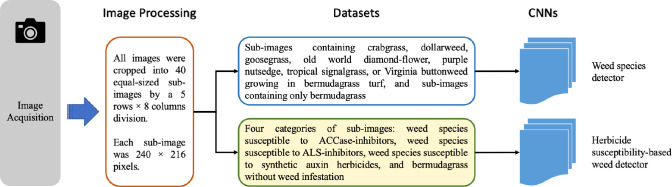
Flow diagram illustrates the sequence of image processing and training and testing the convolutional neural networks.

Images containing crabgrass, dollarweed, goosegrass, old world diamond-flower, purple nutsedge, tropical signalgrass, and Virginia buttonweed growing in bermudagrass turf were selected to constitute the training or testing datasets. Images containing a single weed species were selected for training and testing neural networks. All images were cropped into 40 equal-sized sub-images by a 5 rows × 8 columns division. Each sub-image was 240 × 216 pixels. Sub-images of crabgrass, goosegrass, and tropical signalgrass (**Figure 1**), purple nutsedge (**Figure 2**), dollarweed, old world diamond-flower, and Virginia buttonweed (**Figure 3**) at different growth stages and densities, and sub-images of bermudagrass at varying mowing heights and surface conditions (**Figure 4**) were utilized for training and testing the neural networks. **Figure 5** outlines the sequence diagram of image processing and training and testing the convolutional neural networks for detecting and discriminating individual weed species or weeds susceptible to ACCase-inhibitor, ALS-inhibitor, synthetic auxin herbicides, or bermudagrass without weed infestation (no herbicide).

The convolutional neural networks for detecting and distinguishing weed species were trained utilizing a total of 21,000 true positive sub-images (3,000 sub-images for each weed species) containing crabgrass, dollarweed, goosegrass, old world diamond-flower, purple nutsedge, tropical signalgrass, or Virginia buttonweed growing in bermudagrass turf, while a total of 9,000 sub-images containing only bermudagrass were utilized as the true negative images. To establish the validation or testing dataset, a total of 3,500 sub-images (500 images for each weed species) containing crabgrass, dollarweed, goosegrass, old world diamond-flower, purple nutsedge, tropical signalgrass, or Virginia buttonweed growing in bermudagrass were utilized as the true positive images, while a total of 1,500 sub-images containing only bermudagrass were utilized as the true negative images.

The convolutional neural networks for detecting and distinguishing weeds susceptible to various herbicides were trained using a dataset containing four categories of sub-images: weed species susceptible to ACCase-inhibitors, weed species susceptible to ALS-inhibitors, weed species susceptible to synthetic auxin herbicides, and bermudagrass without weed infestation. To establish the training, validation, or testing dataset, the sub-images containing crabgrass, goosegrass, or tropical signalgrass, the sub-images containing purple nutsedge, the sub-images containing dollarweed, old world diamond-flower, or Virginia buttonweed, and the sub-images containing bermudagrass only were grouped and labeled as ACCase-inhibiting herbicides, ALS-inhibiting herbicides, synthetic auxin herbicides and no herbicide, respectively (**Table 1**).

**Table 1:** The number of sub-images used to establish the training, validation, and testing datasets of the convolutional neural networks.

Dataset	ACCase-inhibiting herbicides			ALS-inhibiting herbicides	No herbicide	Synthetic auxin herbicides		
	Crabgrass	Goosegrass	Tropical signalgrass	Purple nutsedge	Bermudagrass	Dollarweed	Old world diamond-flower	Virginia buttonweed
Training	3000	3000	3000	3000	9000	3000	3000	3000
Validation	500	500	500	500	1500	500	500	500
Testing	500	500	500	500	1500	500	500	500

The convolutional neural networks were trained to detect and discriminate weed species and the sub-images containing weeds susceptible to ACCase-inhibiting herbicides, ALS-inhibiting herbicides, synthetic auxin herbicides, or bermudagrass without weed infestation (no herbicide).

The training and testing of the convolutional neural networks were performed in PyTorch (version 1.8.1) deep learning environment (Facebook, San Jose, California, United States) with an NVIDIA GeForce RTX 2080 Ti graphic processing unit (GPU). Transfer learning seeks to use previously acquired knowledge while addressing one problem and applying it to a different but similar problem (Lu et al., 2015). The convolutional neural networks were pre-trained with the ImageNet dataset to initialize the weights and bias through the transfer learning technology. To ensure fair comparisons among the evaluated deep learning models, default values of hyper-parameters for each neural network were adopted and used (**Table 2**).

**Table 2:** Hyperparameters used for training the convolutional neural networks.

Deep learning architecture	Optimizer	Base learning rate	Learning rate policy	Batch size	Training epochs
DenseNet	SGD	0.001	LambdaLR	16	30
EfficientNet-v2	SGD	0.01	LambdaLR	16	30
ResNet	Adam	0.0001	StepLR	16	30

SGD, stochastic gradient descent.

A binary classification confusion matrix with four conditions, including the true positive (*tp*), false positive (*fp*), true negative (*tn*), and false negative (*fn*), was used to present the training and testing results of the convolutional neural networks. The performances of the convolutional neural networks were evaluated and compared against each other in terms of precision, recall, F_1_ score, and Matthews Correlation Coefficient (MCC).

Precision is the ability of the neural networks to detect the susceptible weed species and was calculated using the *tp* and *fp* (Sokolova and Lapalme, 2009):


(1) $$\begin{array}{c} precision=\;\frac{tp}{tp+fp} \end{array}$$

Recall is the effectiveness of the neural networks to detect the susceptible weed species and was computed using the *tp* and *fn* (Sokolova and Lapalme, 2009):


(2) $$\begin{array}{c} recall\;=\;\frac{tp}{tp+fn} \end{array}$$

The F_1_ score is a commonly used metric for measuring the overall performance of the neural networks, which was defined using the following equation (Sokolova and Lapalme, 2009):


(3) $$\begin{array}{c} F_{1}=\frac{2\times precision\times recall}{precision+recall} \end{array}$$

The MCC is the correlation between ground truth labels and predictions, which was determined using the following equation (Chicco and Jurman, 2020):


(4) $$\begin{array}{c} MCC=\frac{tp\times tn-fp\times fn}{\sqrt{\left(tp+fp\right)\times \left(tp+fn\right)\times \left(tn+fp\right)\times \left(tn+fn\right)}} \end{array}$$

Frames per second (FPS) measures the number of images, also known as frames processed by the neural networks each second. A higher FPS value indicates faster image processing. The FPS value was adopted as a quantitative metric to evaluate the computational efficiency of the neural networks.

## Results

### Detection and discrimination of weed species

When the convolutional neural networks were trained for detecting and distinguishing weed species growing in bermudagrass turf, DenseNet, EfficientNet-v2, and ResNet exhibited excellent performances and achieved high F_1_ scores (≥0.995) and MCC values (≥0.994) in the validation datasets for detecting and distinguishing the sub-images containing dollarweed, goosegrass, purple nutsedge, and the sub-images containing bermudagrass only (**Table 3**). In general, a slight reduction in weed detection performance of all neural networks was observed in the testing datasets compared to the validation datasets.

**Table 3:** Weed species detection and discrimination training results using various convolutional neural networks.

Deep learning architecture	Weed species	Validation dataset				Testing dataset			
		Precision	Recall	F_1_ score	MCC	Precision	Recall	F_1_ score	MCC
DenseNet	Bermudagrass	1.000	0.998	0.999	0.999	0.999	0.999	0.999	0.999
	Crabgrass	0.923	0.940	0.931	0.924	0.920	0.938	0.929	0.921
	Dollarweed	0.998	1.000	0.999	0.999	0.996	0.998	0.997	0.997
	Goosegrass	0.994	0.996	0.995	0.994	0.990	0.996	0.993	0.992
	Old world diamond-flower	0.984	0.994	0.989	0.988	0.980	0.994	0.987	0.986
	Purple nutsedge	0.994	0.998	0.996	0.996	0.996	0.994	0.995	0.994
	Tropical signalgrass	0.937	0.920	0.928	0.921	0.937	0.916	0.926	0.918
	Virginia buttonweed	0.996	0.984	0.990	0.989	0.994	0.978	0.986	0.984
EfficientNet-v2	Bermudagrass	1.000	1.000	1.000	1.000	1.000	0.999	0.999	1.000
	Crabgrass	0.924	0.942	0.933	0.925	0.920	0.938	0.929	0.921
	Dollarweed	1.000	1.000	1.000	1.000	0.998	0.998	0.998	0.998
	Goosegrass	0.998	0.996	0.997	0.997	0.992	0.996	0.994	0.993
	Old world diamond-flower	0.986	0.996	0.991	0.990	0.982	0.994	0.988	0.987
	Purple nutsedge	0.996	0.998	0.997	0.997	0.996	0.994	0.995	0.994
	Tropical signalgrass	0.941	0.922	0.931	0.924	0.937	0.918	0.927	0.919
	Virginia buttonweed	0.996	0.986	0.991	0.990	0.994	0.982	0.988	0.987
ResNet	Bermudagrass	0.999	0.999	0.999	0.998	0.999	0.999	0.999	0.999
	Crabgrass	0.922	0.942	0.932	0.924	0.918	0.938	0.928	0.920
	Dollarweed	1.000	0.998	0.999	0.999	0.998	0.998	0.998	0.998
	Goosegrass	0.998	0.996	0.997	0.997	0.990	0.996	0.993	0.992
	Old world diamond-flower	0.986	0.996	0.991	0.990	0.980	0.994	0.987	0.986
	Purple nutsedge	0.996	0.998	0.997	0.997	0.996	0.994	0.995	0.994
	Tropical signalgrass	0.941	0.918	0.929	0.921	0.937	0.916	0.926	0.918
	Virginia buttonweed	0.992	0.986	0.989	0.988	0.994	0.978	0.986	0.984

For the detection of old world diamond-flower, the recall values of DenseNet in the validation and testing datasets were 0.994, while the precision values were 0.984 and 0.980, respectively, in predicting the correct weed species labels. For the detection of Virginia buttonweed, the precision values of DenseNet were 0.996 and 0.994, respectively, while the recall values were 0.984 and 0.978, respectively. Similar trends were observed in the validation and testing datasets for EfficientNet-v2 and ResNet.

All three neural networks performed poorly at detecting and distinguishing crabgrass and tropical signalgrass growing in bermudagrass turf. Because of low precision and recall values, the F_1_ scores and MCC values of DenseNet, EfficientNet-v2, and ResNet never exceeded 0.918, 0.919, and 0.918, respectively, in the validation and testing datasets. The low F_1_ scores and MCC values indicate that the neural networks are more likely to mistakenly classify crabgrass as tropical signalgrass (or vice versa). This finding could likely attribute to the similarity in plant morphology between crabgrass and tropical signalgrass.

### Detection and discrimination of weeds susceptible to herbicides

No obvious differences were observed among DenseNet, EfficientNet-v2, and ResNet for detecting and distinguishing weeds susceptible to ACCase-inhibitors, ALS-inhibitors, synthetic auxin herbicides, or bermudagrass without weed infestation (no herbicide) (**Table 4**).

**Table 4:** Training and testing results of various convolutional neural networks for detecting and discriminating the sub-images containing weeds susceptible to herbicides, or bermudagrass without weed infestation (no herbicide).

Deep learning architecture	Herbicides	Validation dataset				Testing dataset			
		Precision	Recall	F_1_ score	MCC	Precision	Recall	F_1_ score	MCC
DenseNet	ACCase-inhibiting herbicides	0.999	0.999	0.999	0.998	0.997	0.998	0.997	0.997
	ALS-inhibiting herbicides	0.996	0.998	0.997	0.997	0.996	0.994	0.995	0.994
	No herbicide	0.999	0.999	0.999	0.999	0.999	0.999	0.999	0.999
	Synthetic auxin herbicides	0.999	0.999	0.999	0.998	0.999	0.999	0.999	0.999
EfficientNet-v2	ACCase-inhibiting herbicides	0.999	0.999	0.999	0.999	0.998	0.998	0.998	0.997
	ALS-inhibiting herbicides	0.996	0.998	0.997	0.997	0.996	0.994	0.995	0.994
	No herbicide	0.999	0.999	0.999	0.999	1.000	0.999	0.999	1.000
	Synthetic auxin herbicides	0.999	0.999	0.999	0.999	0.999	1.000	0.999	0.999
ResNet	ACCase-inhibiting herbicides	0.999	0.998	0.998	0.998	0.998	0.995	0.996	0.995
	ALS-inhibiting herbicides	0.996	0.998	0.997	0.997	0.996	0.994	0.995	0.994
	No herbicide	0.998	1.000	0.999	0.999	0.997	0.999	0.998	0.997
	Synthetic auxin herbicides	0.999	0.997	0.998	0.998	0.997	0.997	0.997	0.996

DenseNet, EfficientNet-v2, and ResNet achieved high F_1_ scores and MCC values (≥0.997) with high precision (≥0.996) and recall (≥ 0.997) in the validation datasets. All neural networks had slightly reduced precision and recall values in the testing datasets, but the F_1_ scores and MCC values never fell below 0.994.

These results suggest that convolutional neural networks can reliably detect and distinguish weeds susceptible to particular herbicides. Furthermore, it can be inferred that training the neural networks based on the susceptibility of weed species to herbicides could probably minimize the morphological similarity issue and hence improve weed detection accuracy.

### Inference time of the convolutional neural networks

In addition to the weed detection accuracy, the inference time of the convolutional neural networks is also critical for real-time precision herbicide application. The FPS values of DenseNet, EfficientNet-v2, and ResNet were calculated by averaging the inference time of images from the testing dataset. Since the original images were captured at a resolution of 1,920 × 1,080 pixels, the detection speed with the full images was measured by processing the sub-images (240 × 216 pixels) with a batch size value of 40 (for simultaneously processing 40 sub-images).

All convolutional neural networks, including DenseNet, EfficientNet-v2, and ResNet, had an excellent detection speed (≥77.94fps) when detecting and distinguishing the sub-images with a batch size value of 1 (**Table 5**). DenseNet, with 61.79 full images detected per second, was 31.59 slower than ResNet but noticeably faster than EfficientNet-v2 when setting the batch size value as 40. ResNet demonstrated the fastest inference rate and outperformed the other convolutional neural networks on detection efficiency. However, the slow detection of EfficientNet-v2 may limit its potential applications.

**Table 5:** The inference time of the convolutional neural networks evaluated in the study.

Deep learning architecture	Image type	Resolution	Batch size	FPS
DenseNet	Sub-image	240 × 216	1	103.75
			40	61.79
EfficientNet-v2	Sub-image	240 × 216	1	77.94
			40	38.77
ResNet	Sub-image	240 × 216	1	276.08
			40	93.38

FPS, frames per second.

## Discussion

Deep learning methods for weed detection typically focus on distinguishing weed species, but various weed species with comparable plant morphological features may be found in the turfgrass. Thus, it is difficult for neural networks to achieve high accuracy of detection and discrimination for every weed species. Distinguishing different categories of weed species growing in turf based on their susceptibility to herbicides reduces the complexity of weed detection. By training the neural networks according to the susceptibility of weed species to herbicides, we achieved an excellent performance in weed detection. Moreover, this strategy allows the use of specific herbicides for precision spraying susceptible weeds, thus saving more herbicides.

When training convolutional neural networks for detecting weeds susceptible to herbicides, weed vegetation was grouped and labeled into three categories: weeds susceptible to ACCase-inhibitors, weeds susceptible to ALS-inhibitors, and weeds susceptible to synthetic auxin herbicides. ACCase-inhibitors, such as diclofop-methyl, can be applied in bermudagrass turf for POST control of various grassy weeds, while sethoxydim (cyclohexanedione), another ACCase-inhibitor, is used for POST control of grassy weeds growing in centipedegrass [*Eremochloa ophiuroides* (Munro) Hack.] (Neal et al., 1990; Tate et al., 2021). Synthetic auxin herbicides, such as 2,4-D and mecoprop, are POST herbicides that selectively control broadleaf weeds in bermudagrass turf (Grichar et al., 2008; Reed et al., 2013). ALS-inhibitors (e.g. halosulfuron, imazaquin, and trifloxysulfuron-sodium) can effectively control nutsedge weeds. However, it should be noted that certain ALS-inhibitors, such as halosulfuron and trifloxysulfuron-sodium, can also suppress or effectively control broadleaf weeds (McElroy and Martins, 2013). In this context, broadleaf and nutsedge weeds could be grouped into the same category when training the neural network for precision spraying the ALS-inhibitors that are effective for controlling both broadleaves and nutsedges growing in bermudagrass turf.

Deep learning neural networks, including image classification and object detection neural networks, can be developed and potentially integrated into the machine vision sub-system of a smart sprayer. Nevertheless, it should be noted that image classification neural networks alone do not localize weeds on the input images. Consequently, when utilizing image classification neural networks for weed detection, a smart sprayer likely generates a considerably larger spraying output area than the area covered by weeds. In the present work, localizing weeds with image classification neural networks could be realized by cropping the input image into multiple grid cells (sub-images) and identifying the grid cells containing weeds.

In the present study, original images (1,920 × 1,080 pixels) were divided into 40 grid cells (sub-images with a resolution of 240 × 216 pixels) for training and testing the image classification neural networks. Spraying areas can be localized by detecting if the grid cells contain weeds. When developing a precision spraying system, custom software can be programmed to generate grid cell maps on the input images and realize precision herbicide application by detecting if the grid cells contain weeds susceptible to the herbicides. To realize precision herbicide spraying, a binary (on/off) input command can be implemented *via* a nozzle control system to turn off the spray nozzles over the weed-free cells while the nozzles corresponding to the grid cells containing weeds need to be turned on.

While the convolutional neural networks achieved high classification rates for detecting and distinguishing weeds susceptible to herbicides, it should be noted that when weeds susceptible to different herbicides are grown too close or occluded, the neural networks would not effectively distinguish weed categories based on their susceptibility to the herbicides because the grid cell contains multiple targets. Although such a case may result in missed detection, this is hardly an issue in field applications because the weed infestation zone has been detected, and one of the herbicides will be sprayed onto the susceptible weeds.

It was reported that the training image size could significantly affect the reliability of image classification neural networks for weed detection (Zhuang et al., 2021; Yang et al., 2022b). For example, Zhuang et al. observed increased classification accuracy (high recall values) with AlexNet and VGGNet when they were trained with images of 200 × 200 pixels than 300 × 300 or 400 × 400 pixels; however, increasing training image quantities diminished the differences in detection accuracy (Zhuang et al., 2021). In the present study, each sub-image (240 × 216 pixels) represented a physical size of 10 cm × 9 cm. When the convolutional neural networks are integrated into the machine vision sub-system of smart sprayers for precision herbicide application, the nozzles should generate the same or slightly larger spraying outputs to cover the grid cells. An additional investigation is needed to investigate the implications of training image sizes and quantities on the performances of neural networks for weed detection in turf.

## Conclusions

The present research demonstrated the reliability and effectiveness of using convolutional neural networks to detect and distinguish weeds growing in bermudagrass turf based on their susceptibility to herbicides. All convolutional neural networks, including DenseNet, EfficientNet-v2, and ResNet achieved excellent F_1_ scores (≥ 0.995) and MCC values (≥ 0.994) in the validation and testing datasets to detect and distinguish weeds susceptible to ACCase-inhibitors, ALS-inhibitors, and synthetic auxin herbicides, or bermudagrass turf without weed infestation (no herbicide). In addition, DenseNet, EfficientNet-v2, and ResNet had an excellent detection speed (≥77.94fps) when detecting and distinguishing the sub-images with a resolution of 240 × 216 pixels. For detecting the original/full images (1,920 × 1,080 pixels), ResNet demonstrated the fastest inference rate and outperformed the other convolutional neural networks on detection efficiency (93.38fps). Effective detection and discrimination of weeds susceptible to herbicides enable the smart sprayer to spray particular herbicides to control susceptible weeds, thereby significantly reducing herbicide input. Based on the high-level performance, we conclude that the proposed method is highly suitable for integrating into the machine vision sub-system of smart sprayers for the precision control of weeds while growing in turf.

## Data availability statement

The original contributions presented in the study are included in the article/supplementary material. Further inquiries can be directed to the corresponding author.

## Author contributions

XJ conceived the research ideas and designed the experiments under the guidance of YC and JY. TL, PM, and JY collected the data and conducted the data analysis. XJ drafted the manuscript. PM, YC, and JY edited and revised the manuscript. All authors contributed to the article and approved the submitted version.
